# The Effects of Recent Polarized Elections on Mental Health

**DOI:** 10.1111/1468-0009.70086

**Published:** 2026-05-01

**Authors:** MICHAEL E. SHEPHERD, BETHANY ALBERTSON

**Affiliations:** ^1^ University of Michigan; ^2^ University of Texas at Austin

## Abstract

**Policy Points**

- Researchers investigate how recent elections in the United States have influenced mental health, especially among political‐ and policy‐based election losers.
- The previous two presidential elections worsened the self‐reported mental health of Americans on average.
- Likely partisan election losers and those who had the most to lose in terms of health policy were even more likely to have their mental health affected by the results of elections.
- As American politics has become increasingly polarized and the perceived stakes of elections have loomed larger in recent years, elections have become a source of worsening mental health for Americans.

**Context:**

Politics is increasingly important to many Americans. Yet little is known about how the increasing centrality of politics affects Americans’ mental health. This work aimed to evaluate how recent polarized elections have influenced Americans’ mental health.

**Methods:**

To investigate this question, we compared online search interest in politically related mental health issues and self‐reported mental health data. Analyses explored changes before and after election days in 2020 and 2024. The two outcome variables were aggregate Google search interest in politics‐related mental health issues and individual responses to the following item from the Behavioral Risk Factor Surveillance System (BRFSS): ʻʻNow thinking about your mental health, which includes stress, depression, and problems with emotions, for how many days during the past 30 days was your mental health not good? With BRFSS, we compared differential changes for likely Democrats and Republicans using multiple proxy measures and for those with health policy interest in the election.

**Findings:**

The 2020 and 2024 presidential elections substantially increased interest in politics‐related mental health issues online. The 2020 election led to just under 0.2 additional days of poor mental health (*P* < .05), and the 2024 election led to just under 0.5 additional days of poorer mental health (*P* < .05). Likely losing partisans and those who stood to lose out from Trump's reelection in terms of health policy were found to drive most of this relationship, with just under 1 full additional day of poorer mental health for each group.

**Conclusions:**

The stakes of elections in this polarized era of American politics are worsening the mental health of Americans. Additional resources may be necessary to allow therapists and clinicians to navigate additional care‐seeking surrounding and following elections.

## Introduction

Health and politics are deeply intertwined and have become increasingly so during the current era. As American politics has polarized in recent years, the public has viewed elections as having higher personal stakes.^1^ These stakes have been especially high in recent elections. For instance, in each of the past three elections, President Trump has campaigned on and later enacted policies that restrict access to health care and jeopardize the health of Americans, especially those who are more vulnerable, whereas Democratic candidates have pushed for major safety net expansions.^2^, ^3^ As a result, the personal, political, and policy implications of recent elections have increased dramatically. However, too little is known about how Americans’ reactions to recent elections or the stakes of elections and a polarized political environment more generally influence individuals’ mental health.

Traditionally, scholarship on the connections between health and politics has fallen into two categories: 1) how health experiences influence participation in politics and 2) how political dynamics influence health policy or population health outcomes. In terms of the former, considerable scholarship has documented how personal health ratings, familial health experiences, the loss of health care access, and factors such as disability status can also influence one's ability to turn out to vote in elections.^4^, ^5^, ^6^, ^7^ In terms of the latter, scholars have demonstrated that political parties and health‐related political interest groups, such as the American Medical Association and physicians, are critical for understanding the trajectory of population health outcomes and the adoption of health policies.^8^, ^9^, ^10^, ^11^

Quite clearly, political dynamics are critical for shaping health at the level of policy, and health is critical for shaping individual politics. However, much less is known about how individual reactions to or exposure to increasingly polarized politics can shape an individual's health directly. This gap is especially relevant for mental health. Politics can be emotionally stimulating as well as taxing. Although significant scholarship has documented the myriad of ways in which politics evokes emotional responses and emotional processes influence political behavior,^12^, ^13^, ^14^ researchers have developed only a limited understanding of the implications of these political experiences on individuals’ underlying mental health.^15^ Whereas political issues such as terrorism, climate change, and environmental disasters have been shown to induce significant anxiety or fear,^13^, ^16^, ^17^, ^18^ broader partisan politics, and polarization influential for sparking feelings of rage or anger,^19^ and whereas large ideological differences have been found in reported happiness,^20^, ^21^, ^22^ scholars have developed only a limited understanding of how such emotional responses to electoral politics are related to individuals’ underlying mental health.

The closest previous research to this study demonstrated that Americans who share the demographic backgrounds of the losing presidential and vice presidential candidates report worse mental health following the election.^15^ Moreover, partisans in recent years have reported more sadness following election losses.^23^ Additionally, the 2016 Brexit vote in the United Kingdom produced a shift in the political and economic outlook, as well as an increase in antidepressant prescriptions for many British citizens.^24^, ^25^ Drawing on this literature, we ask whether and how individual reactions to polarized political conflict influence Americans’ mental health, especially among those who stand to lose the most from the election itself.

## Methods

To explore this question, we first gathered and aggregated Google search trend scores for a series of election‐ and politics‐related mental health searches. The searches were performed using variations of the terms elections, politics, and/or political alongside variations of the terms stress, anxiety, therapy, therapist, depressed, worry, and/or trauma. The data provide a sense of the public salience and interest in election‐ and politics‐related mental health concerns. The Google Search term interest score for each phrase during each month was used to create an aggregate measure of monthly Google Search interest by calculating the sum of each search phrase score for that month. The data range from a monthly low Google search interest of zero, with about 26% of months having zero search interest, to a high of 1,365 (November 2024). The median aggregate search interest score across the searches was 52, and the mean was 131.4. With these data, we constructed plots of monthly trends to highlight the importance of presidential elections in driving search interest in politics‐related mental health worries. In Appendix 1, we provide the results of ordinary least squares (OLS) regression methods that regress monthly Google search interest on dummy variables for the Novembers of 2004, 2008, 2012, 2016, 2020, and 2024, as well as month and year fixed effects, which rule out year‐to‐year shocks to the data as well as heightened average searches for given months (e.g., if more search interest was observed in all Decembers).

In addition, we used data from the Behavioral Risk Factor Surveillance System (BRFSS) surveys from 2020 and 2024. Each month, the BRFSS completes interviews of just under 40,000 Americans via landline and cell phone about their personal health experiences and evaluations. In this study, the dependent variable comprised responses to the following BRFSS survey item: “Now thinking about your mental health, which includes stress, depression, and problems with emotions, for how many days during the past 30 days was your mental health not good?” An individual's response to this question is recorded as a number between 1 and 30 for the number of poor mental health days in the preceding 30‐day period. The average response from 2020 and 2024 was 11.1 days.

Our interest in this study was in whether polarized political conflict negatively influences mental health. There is little question that American political parties and politics have polarized in recent years.^1^ Elected officials and political candidates from the Democratic and Republican Parties enact and campaign on increasingly distinctive policy agendas, Democrats and Republicans in the mass public increasingly view the opposing party with derision, and the mass public generally has reported increasingly high policy stakes in recent elections.^1^, ^26^ However, estimating the effect of increasingly polarized political conflict on mental health is empirically difficult. Whereas overall mental health outcomes have worsened and mental health service utilization has increased over the same years that political polarization has increased, many other potentially confounding factors have unfolded simultaneously (e.g., climate change and rising income inequality).^13^, ^26^, ^27^

As a result, we opted for a quasi‐experimental design that exploits the exogenous timing of elections as a source of increased public focus on and exposure to polarized political conflict. Americans tend to pay the most attention to and express the most interest in politics near presidential elections.^28^, ^29^ Moreover, scholars have shown that elections tend to increase polarization and raise the salience of partisan conflict generally.^30^, ^31^, ^32^, ^33^ Thus, we used the exogenous timing of elections to measure the effect of polarized political conflict both in general and among different groups of voters. This relationship was captured as a dummy variable for interviews that were on or that followed election day in November 2020 and November 2024, as well as those that followed in the corresponding Decembers, to capture the day of and the period following the election (1) or for interviews that were conducted before the election (0). Roughly 17% of the respondents were interviewed between election day and the end of December in 2020 and 2024. The baseline model in each year consisted of an OLS regression model of the mental health outcome on the post‐election dummy variable, as well as state fixed effects. For all regression analyses, we used the survey weights provided by the BRFSS.

To probe mechanisms related to the influence of polarized political conflict on mental health, we allowed the effect of elections to vary by either the respondents’ likely partisan leanings or the respondents’ likely personal policy consequences resulting from recent polarized elections. Specifically, we used two models with differing proxy operationalizations of the mechanism of partisan reactions to polarized political conflict before and after the 2020 and 2024 elections. To capture these partisan reaction effects, we allowed the estimated influence of the election in one model to vary by whether the respondent resided in a Republican state (1), that is, in a state that was won by the Republican ticket in every presidential election between 2016 and 2024 and was not considered a battleground state throughout the period, or did not (0). This was done by including a dummy variable for these states, as well as a statistical interaction with the post‐election dummy variable. These states are listed in Appendix 2. In total, about 39% of respondents resided in these Republican states. For this analysis, the state fixed effect was omitted because the Republican state status does not vary within a state over the course of a year. For the second model, we allowed the estimated influence of the election to vary depending on whether the respondent was someone considered most likely to be a Democrat. Specifically, women, people of color, and urban residents are substantially more likely to be Democrats than Republicans.^34^, ^35^, ^36^, ^37^ Indeed, data from the Cooperative Election Study cumulative file from the years 2006‐2024 show that roughly 85% of urban respondents who identified as women of a race or ethnicity other than White voted for the Democratic candidate in the most recent election.^38^ A respondent was thus considered most likely to be a Democrat if they were a woman of color living in a metropolitan area (1) versus otherwise (0). Once again, this term was included along with its statistical interaction with the post‐election dummy variable. In total, just over 10% of respondents were coded with the proxy measure for “most likely to be Democrats.” For this second analysis, we did include state fixed effects. Importantly, both of these models considered proxy measures of partisanship and not partisanship itself. For example, the “most likely Democrat” observations may be overestimating the effect of partisanship by focusing on a group that may respond to the results of the election most strongly. However, in the case of the observations of “Republican states,” we suspect that we may be underestimating the effects of partisanship because of the heterogeneous makeup of even the most partisan states. Thus, the two models likely provide a bounded estimate of the influence of a partisan election win or loss.

In addition to purely partisan responses, polarized political conflict may influence an individual's mental health through the personal policy consequences resulting from or implied by election outcomes. In the current era of polarized politics, elections have larger policy stakes. Although we were limited in the number of policy‐relevant variables available in the BRFSS survey, we were able to capture whether a person received their health insurance from Medicaid, from Medicare, or through the Affordable Care Act (ACA), and health insurance is a high‐stakes policy on which the two parties differ sharply. Given Donald Trump's repeated attacks on and attempts to repeal the Affordable Care Act alongside his cuts to other social safety net programs during his first and second terms and his proposals to continue these efforts on the campaign trail in 2024,^3^, ^39^, ^40^, ^41^ as well as Democratic candidates’ and President Biden's support of the ACA, Medicaid expansion, and Medicare prescription drug cost negotiations, the policy ramifications of recent elections in this area have been especially clear for affected voters. Further, scholars have shown that those who benefit from or are connected to Medicaid, Medicare, and the ACA favor their expansion, resist their contraction, and engage in politics in ways that reflect these concerns at election time.^41^, ^42^, ^43^

Thus, one may expect those who receive their health insurance from these sources to respond with worse mental health following Trump's victory in 2024 and better mental health following Biden's victory in 2020. Respondents who obtained their health insurance from Medicaid, Medicare, or these other specific state‐sponsored programs were coded as receiving government insurance (1), whereas those obtaining health insurance from other sources were coded as 0. Roughly 25% of respondents obtained their health insurance from the mentioned government sources.

Table 1 provides summary statistics for these variables.

**Table 1:** Summary Statistics for Key Variables from the BRFSS

Variable	*N*	Mean	Std Dev	Min	Max
Poor mental health days	317,927	11.096	10.124	1	30
Election	859,628	0.167	0.373	0	1
Most likely Democrat	859,628	0.101	0.301	0	1
Republican state resident	845,184	0.386	0.487	0	1
Has government health insurance	852,646	0.253	0.435	0	1

Abbreviations: BRFSS, Behavioral Risk Factor Surveillance System; max, maximum; min, minimum; std dev, standard deviation.

Data derived from BRFSS 2020 and 2024.

## Results

**Figure 1:**
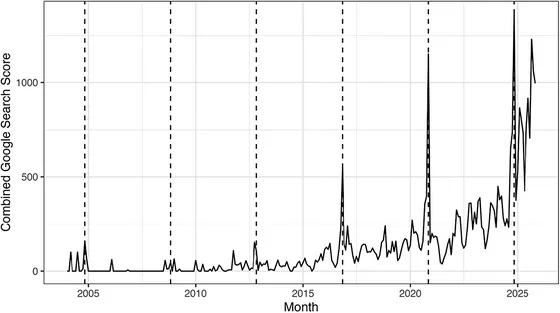
Political Anxiety Google Search Trends, 2004‐2025 Data from Google Search Trends. Shown are combined Google Search Trend interest scores for election stress, political stress, election anxiety, political anxiety, political therapist, politics therapist, political therapy, politics therapy, depressed election, depressed politics, depressed political, anxiety politics, anxiety political, stress politics, stress political, stress election, worrying election, politics mental health, election mental health, therapist election, therapy election, anxious election, political trauma, and election trauma.

Figure 1 displays the monthly Google search score for each of these term variations from 2004 through 2025. The months of the 2004, 2008, 2012, 2016, 2020, and 2024 presidential elections are indicated with dashed lines. The plot reveals that Google searches for election‐ and politics‐related mental health concerns have increased substantially over the past decade. The average search interest score was 36 in 2004, 10 in 2008, and 41 in 2012, indicating stable concerns about politics‐related mental issues until recently. During the three most recent elections, the average Google search interest score was 141 in 2016, 288 in 2020, and 468 in 2024. The values in November of 2016, 2020, and 2024 were the largest spikes in each of those years. Following the 2024 election, Google searches for politics‐related mental health terms continued to climb. In Figure A2 in Appendix 2, one can see that related spikes and patterns also held for searches coming from Republican states, reducing the possibility that partisan differences in mental health care utilization and concerns can explain the results.

**Figure A1:**
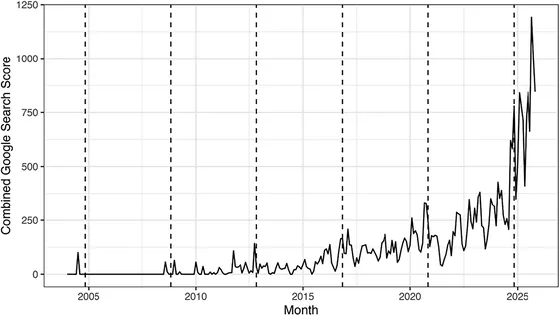
Results of Political Mental Health Searches Omitting the Word “Election”

**Figure A2:**
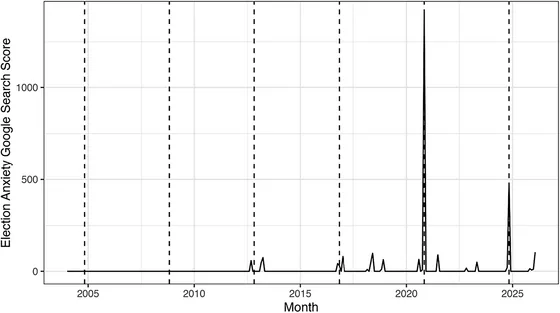
Election Anxiety Mental Health Searches in Republican States

For the data and OLS regression methods used in this work, Table A4 in Appendix 1 shows that, after month and year fixed effects had been taken into account, the 2016, 2020, and 2024 presidential elections were associated with increased politics‐related mental health searches. From 2016 to 2020, the post‐election spike doubled in size. From 2020 to 2024, the post‐election spike increased by nearly 12%. One may be concerned that these shifts, especially those corresponding to elections, might have been driven by searches that specifically included the word “election.” However, in Appendix 2, Figure A1 shows that similar trends of increasing political anxiety‐related searches were observed even after searches that mentioned elections directly were excluded and that both the 2020 and 2024 elections exhibited election‐specific spikes.

**Figure 2:**
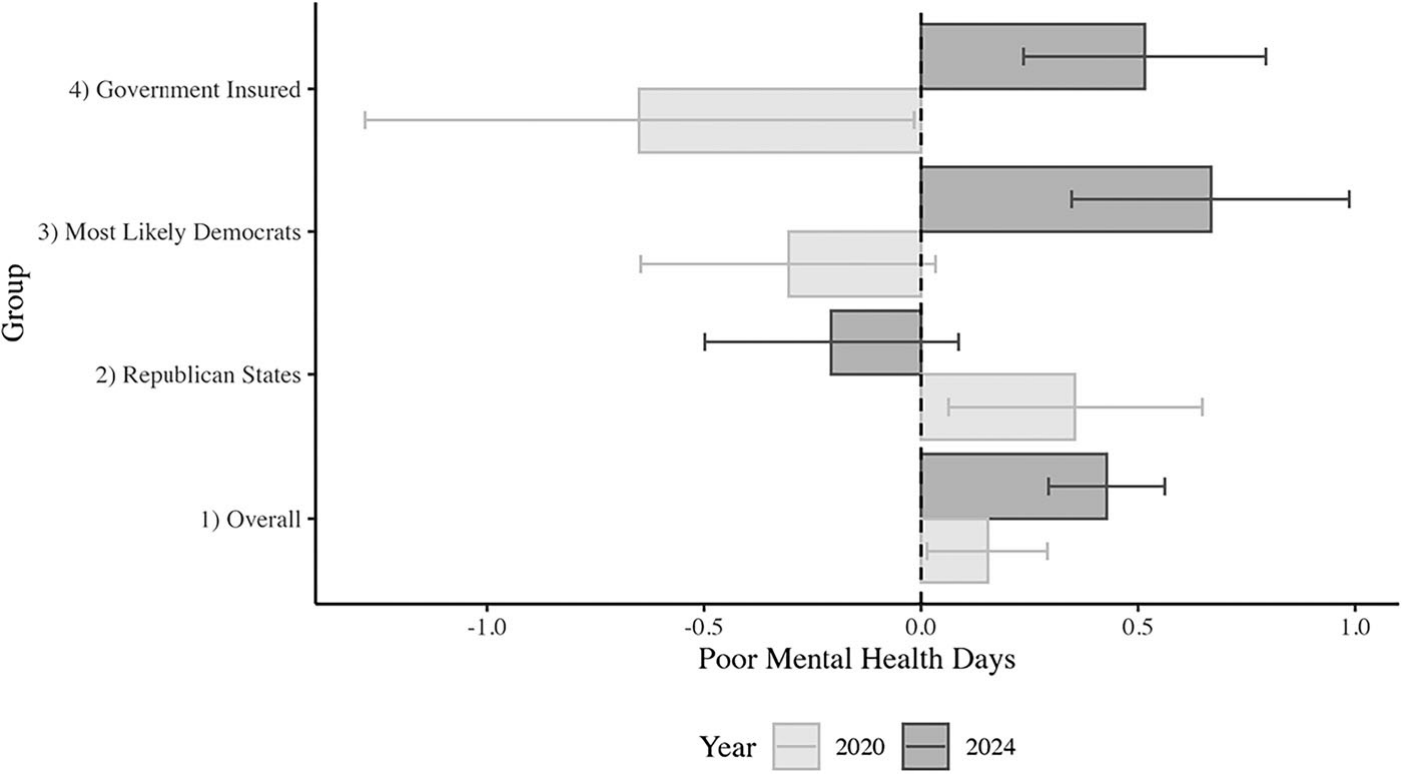
Regression Coefficients for Changes in Mental Health Post‐Election, 2020 and 2024 Results from separate OLS regression analyses. Parameter estimates are provided in bars with 95% confidence intervals. Results obtained using 2020 data are in light gray, and those obtained using 2024 data are in dark gray. “Overall” indicates estimated post‐election differences for the full sample. “Republican States” indicates the difference in mental health for respondents in Republican states before and after the election through statistical interaction. “Most Likely Democrats” represents the differences in mental health for those most likely to be Democrats before and after the election through statistical interaction. “Government Insured” presents the difference in self‐reported mental health for respondents who obtained their insurance from Medicaid, Medicare, or another state‐sponsored program before and after the election through statistical interaction.

Figure 2 presents the primary results of the analyses of the BRFSS survey data. The full results are available in Tables A1, A3, and A5 in Appendix 1. The figure provides coefficient estimates for 2020 in light gray and for 2024 in dark gray, along with associated 95% confidence intervals for the estimates. Results labeled “Overall” provide the average post‐election change from the baseline model. Results labeled “Republican States” represent the Republican state interaction term from the first model, those labeled “Most Likely Democrats” represent the most likely Democrats interaction term from the second model, and those labeled “Government Insured” represent respondents who obtained their insurance fromS Medicaid, Medicare, or a state‐sponsored health insurance program.

Overall, we found that respondents interviewed in the month of and in the month following the 2020 and 2024 elections experienced significantly higher rates of poor mental health. In 2020, respondents experienced 0.15 additional days per month of “not good” mental health. In 2024, respondents experienced 0.4 additional days per month of poorer mental health. This 2024 increase represents a 4% increase in poor mental health relative to the 2024 average and is more than twice the size of the 2020 increase, indicating growing mental health consequences of recent elections, especially following Trump's 2024 victory. One may be concerned that the size of this spike has been misestimated because of the inclusion of individuals who were interviewed in the weeks immediately following the election. This possibility was probed by omitting all interviews that occurred within 1 and 2 weeks of the election (see Appendix 2, Table A6). If anything, the results indicate that the sizes of the post‐election spikes were somewhat underestimated.

Although these results are consistent with election‐driven changes in mental health, one may be concerned that the detected post‐election shifts were seasonal rather than electoral. Indeed, winter weather is associated with worsening mental health for many, often resulting from seasonal affective disorder.^44^ To assuage such concerns, we conducted a placebo analysis using the 2023 BRFSS data. In an off‐year such as 2023, similar post‐election shifts should not be observed. The placebo treatment considered all November and December responses from 2023 for the placebo treatment. Table A2 in Appendix 1 reports the results of this analysis. Although a marginal increase in poor mental health was found for November and December, the result was not statistically significant and was substantially smaller than the post‐election spikes observed in 2020 and 2024. Thus, one can be reasonably confident that the observed shift was not a yearly seasonal change.

We further found that clear partisan and policy‐relevant mechanisms were driving these changes. In 2020, residents of Republican states, those more likely to be losers of the 2020 election given the victory by Democrat Joe Biden, experienced just under 0.4 additional days of poor mental health, more than twice the overall estimate. In contrast, the mental health of respondents in the Republican states who were on the winning side of the 2024 election following Republican Donald Trump's victory experienced 0.2 *fewer* days of poor mental health, although this estimate narrowly missed traditional standards of statistical significance (*P* = .16).

Following a Democratic victory in the 2020 election, those proxied as most likely to be Democrats reported somewhat better mental health, with a decrease of 0.3 poor mental health days. In contrast, the group of those proxied as most likely to be Democrats experienced just under 0.7 additional days of poor mental health following the 2024 presidential election loss, nearly two times larger than the average shift observed overall after the 2024 election. Importantly, these results reveal opposing and reversing *within‐party* shifts following recent elections. The within‐party changes before and after a single election rule out concerns that Democrats’ baseline higher likelihood of reporting worse mental health and exhibiting greater utilization of mental health services than Republicans is driving the result.^20^, ^45^ The roughly equally opposing and reversing patterns for proxied Democrats and Republicans after elections lend further evidence of a partisan reaction mechanism.

Support can also be seen for a mechanism of those with the most health policy vulnerability from election outcomes responding more strongly than others, reversing the expected directions if polarized election results were leading to unique shifts. In 2020, those who were receiving their health insurance from government sources experienced just under a 0.7‐day reduction in days of poor mental health in response to Democrats winning the White House, consistent with a perceived reduction in the plausibility of adverse policy changes relative to a second Trump term and an increased likelihood of more favorable policies under Biden. The exact opposite pattern was apparent following Trump's victory in 2024. Relative to prior months in that year, those who were receiving their health insurance from government sources experienced a 0.5‐day increase in the number of poor mental health days. This pattern is consistent with those who expected to experience and ultimately did experience adverse policy changes under Trump responding most negatively to his victory.

One may be curious whether the partisan or policy mechanism was associated with longer‐lasting effects on mental health. This possibility was explored with the 2024 data by limiting the sample of post‐election respondents in multiple increments to respondents interviewed during weeks further from the election date and rerunning the regression analysis (see Appendix 2, Tables A7 and A8). Post‐election relationships for partisans or policy‐concerned respondents that persisted or increased over this time would be indicative of a more durable shift in mental health. Post‐election spikes in worsening mental health that ceased to be significant among respondents of a given subgroup as election day faded in memory would be reflective of a short‐term blip in mental health. In fact, the partisan mechanism was found to be more consistent with a short‐term blip, as the mental health of those proxied to most likely be Democrats was no longer significant when focusing on those interviewed more than three weeks after the election. However, the policy concerns mechanism persisted for at least three weeks after the election and, if anything, showed signs of increasing importance over time.

Some may also be interested in partisan differences in mental health responses among those who experienced policy‐relevant consequences as a result of the elections. For 2024, one may expect that respondents most likely to be Democrats who also had policy‐specific consequences would exhibit a larger spike in poor mental health than either those who were most likely to be Democrats or those who had policy‐specific consequences in isolation. We tested this possibility by estimating changes in poor mental health as a function of the election dummy variable, the proxy variable for most likely to be a Democrat, the government insurance dummy variable, the interactions between these terms, and a triple interaction among the three terms alongside state fixed effects. Table A9 in Appendix 2 presents the results of this analysis. The results of this regression imply that those proxied to most likely be Democrats alone experienced 0.395 (0.207) additional days of poor mental health following the election, those who stood to lose in policy terms due to insurance status alone experienced an estimated additional 0.416 (0.162) days in poor mental health, and those who were both proxied to be Democrats *and* to face more severe policy consequences experienced an additional 1.47 (0.345) days of poor mental health.

## Discussion

Recent elections have significantly influenced Americans’ mental health. Those on the losing side of the 2020 and 2024 elections in terms of proxied partisanship experienced significantly worse mental health in the immediate aftermath of the election, whereas those on the winning side of these elections experienced better mental health. Additionally, those who faced the largest potential health policy consequences from a Trump victory responded to his 2020 loss with improved mental health and to his 2024 victory with worse mental health. We attribute these shifts to the overall increasing polarization of the two parties and their policies, which have heightened relevance immediately following elections.

These findings have significant implications for health policy and the provision of behavioral health care. In terms of policy, these findings imply that the current American political environment necessitates more resources to be devoted to behavioral health, especially surrounding elections and especially for those who perceive significant personal policy‐related consequences as a result of the election outcome. As feelings of anxiety and depression spike near elections and reactions to the polarized politics worsen American health, targeted interventions may be required to reduce the health consequences of recent political developments, events, and crises.

Additionally, these findings point to the increasing need for expanded “structural competency” training for psychiatrists and other behavioral health professionals.^46^ Scholars of behavioral health have increasingly recognized the importance of better incorporating knowledge of how social structures, such as policies and institutions, influence clinical experiences for both patients and physicians.^46^ Indeed, psychiatrists, therapists, and other behavioral health professionals in the United States have noted that politics has become increasingly important for therapy provision in recent years.^47^, ^48^ These findings demonstrate just how critical advancements in politics‐related structural competency training in behavioral health are for the future of managing the mental health of Americans.

Yet, because most Americans do not access mental health care, clinical solutions alone are insufficient. Developing accessible, evidence‐based best practices for managing politically induced stress, through public health campaigns, community organizations, or workplaces, may be equally critical for addressing the broader mental health consequences of contemporary American political life. Future research might also examine discrete political shocks as natural experiments in this vein; the COVID‐19 pandemic and the January 6th Capitol attack, for instance, offer compelling potential case studies of how acute political trauma propagates through the population's mental health.

## Conclusions

The polarization of American politics and recent elections is increasingly influential in shaping the mental health of Americans. Individuals on the losing side of elections in terms of policy and partisan politics face heightened rates of poor mental health. Partisan politics and elections must be more actively considered as part of behavioral health training and provision.

## Funding/Support

No funding support was utilized in this research.

## Conflict of Interest Disclosures

No conflicts of interest were reported.

## Appendix 1

### Full Regression Results and Coding Decisions

Appendix 1 includes the full regression output for all analyses included in the main text. Table A1 presents the output for the 2024 results. Table A2 summarizes the results for the 2023 placebo analysis. Table A3 lists the output for the 2020 results. Table A4 presents the full results for the regression analysis of Google search trends. Table A5 provides the full results for 2020 and 2024 for those who received their health insurance from government sources.

For the purposes of the state‐based partisanship proxy, states that voted for the Republican presidential ticket in every presidential election from 2016 to 2024 and that were not considered battleground states in any of the recent presidential elections were designated as Republican states. These 22 states were Alabama, Arkansas, Idaho, Indiana, Iowa, Kansas, Kentucky, Louisiana, Mississippi, Missouri, Montana, Nebraska, North Dakota, Ohio, Oklahoma, South Carolina, South Dakota, Texas, Tennessee, Utah, West Virginia, and Wyoming.

**Table A1:** Regression Analysis of BRFSS Poor Mental Health Days, 2024

OLS	DV: Number of Days of “Not Good” Mental Health in 2024		
State fixed effects	Yes	Yes	No
Survey weight	Yes	Yes	Yes
Post‐election	0.428** (0.068)	0.292** (0.076)	0.495** (0.080)
Most likely Democrat		0.189** (0.064)	
Republican state resident			0.563** (0.055)
Post‐election × most likely Democrat		0.667** (0.166)	
Post‐election × Republican state resident			−0.206 (0.149)
*N*	179,605	179,605	177,656
Adjusted *R*‐squared	0.005	0.005	0.001

Abbreviations: BRFSS, Behavioral Risk Factor Surveillance System; DV, dependent variable; OLS, ordinary least squares.

**P* < .1; ***P* < .05.

**Table A2:** Regression Analysis of Poor Mental Health Days, 2023 Placebo

OLS	DV: Number of Days of “Not Good” Mental Health in 2023
State fixed effects	Yes
Survey weights	Yes
Election	0.130 (0.068)
*N*	168,189
Adjusted *R*‐squared	0.006

Abbreviation: DV, dependent variable; OLS, ordinary least squares.

**P* < .1; ***P* < .05.

**Table A3:** Regression Analysis of BRFSS Poor Mental Health Days, 2020

OLS	DV: Number of Days of “Not Good” Mental Health in 2020		
State fixed effects	Yes	Yes	No
Survey weight	Yes	Yes	Yes
Post‐election	0.153** (0.071)	0.214** (0.079)	−0.070 (0.087)
Most likely Democrat		0.233** (0.078)	
Republican state resident			0.864** (0.065)
Post‐election × most likely Democrat		−0.306** (0.173)	
Post‐election × Republican state resident			0.355** (0.149)
*N*	138,322	138,322	136,741
Adjusted *R*‐squared	0.008	0.008	0.002

Abbreviations: BRFSS, Behavioral Risk Factor Surveillance System; DV, dependent variable; OLS, ordinary least squares.

**P* < .1; ***P* < .05.

**Table A4:** Regression Analysis of Election Date and Political Mental Health Concern Google Search Interest

OLS	DV: Google Search Score
Month fixed effects	Yes
Year fixed effects	Yes
2024 election month or after	459.480*** (72.197)
2020 election month or after	411.680*** (72.197)
2016 election month or after	222.480*** (72.197)
2012 election month or after	−6.020 (72.197)
2008 election month or after	−7.920 (72.197)
2004 election month or after	68.780 (72.197)
*N*	263
Adjusted *R*‐squared	0.827

Abbreviation: DV, dependent variable; OLS, ordinary least squares.

****P* < .01.

**Table A5:** Regression Analysis of BRFSS Poor Mental Health Days by Health Status

	DV: Number of Days of “Not Good” Mental Health	
**OLS**	**2020**	**2024**
State fixed effects	Yes	Yes
Survey weight	Yes	Yes
Election	0.183** (0.073)	0.242** (0.082)
Has government insurance	1.240** (0.162)	2.236** (0.055)
Election × has government insurance	−0.649** (0.323)	0.515** (0.143)
*N*	138,048	177,806
Adjusted *R*‐squared	0.009	0.017

Abbreviations: BRFSS, Behavioral Risk Factor Surveillance System; DV, dependent variable; OLS, ordinary least squares.

**P* < .1; ***P* < .05.

## Appendix 2

### Robustness Checks

Appendix 2 presents various robustness checks of the main results. The robustness of the Google search trends data was probed first by omitting all searches that used the word “election” (Figure A1) and then also by looking at election‐specific searches in Republican states (Figure A2). Of the 22 “Republican states,” 15 (about 70%) had more than one month of “election anxiety” Google search interest from 2016 to 2024 to gauge overall trends. Data from these 15 states were used for Figure A2. Table A6 demonstrates the robustness of the overall election spike in poor mental health when the sample used in analysis was limited by omitting all observations within 1 month prior to the election, as well as all interviews conducted within 1 and 2 weeks following the election. As reported in Table A7, the longevity of the effect of the 2024 election on those receiving their insurance from government sources was probed by omitting all interviews that occurred within 1, 2, and 3 weeks following the election. Table A8 provides the results of a similar robustness check for the effect of the election among respondents considered “most likely to be Democrats.” Finally, Table A9 presents the variations in the effect of the election for those who were most likely to be Democrats and/or who received their health insurance from government sources.

**Table A6:** Regression Analysis of BRFSS Poor Mental Health Days, 2024, Alternative Windows

OLS	DV: Number of Days of “Not Good” Mental Health in 2024		
State fixed effects	Yes	Yes	No
Survey weight	Yes	Yes	Yes
Post‐election	0.433** (0.068)	0.451** (0.072)	0.545** (0.079)
Omitted observations	All interviewed within 1 month prior to election day	All interviewed within 1 week of the election	All interviewed within 2 weeks of the election
*N*	166,257	174,534	170,154
Adjusted *R*‐squared	0.005	0.005	0.005

Abbreviations: BRFSS, Behavioral Risk Factor Surveillance System; DV, dependent variable; OLS, ordinary least squares.

**P* < .1; ***P* < .05.

**Table A7:** Regression Analysis of BRFSS Poor Mental Health Days by Health Status Alternative Bandwidths

OLS	DV: Number of Days of “Not Good” Mental Health		
State fixed effects	Yes	Yes	Yes
Survey weight	Yes	Yes	Yes
Election	0.259** (0.088)	0.294** (0.096)	0.233** (0.107)
Has government insurance	2.244** (0.055)	2.242** (0.055)	2.240** (0.055)
Election × has government insurance	0.488** (0.152)	0.648** (0.166)	0.581** (0.184)
Omitted observations (weeks since election)	Within 1 week	Within 2 weeks	Within 3 weeks
*N*	172,789	168,461	164,365
Adjusted *R*‐squared	0.017	0.017	0.016

Abbreviations: BRFSS, Behavioral Risk Factor Surveillance System; DV, dependent variable; OLS, ordinary least squares.

**P* < .1; ***P* < .05.

**Table A8:** Regression Analysis of BRFSS Poor Mental Health Days by Likely Party Alternative Bandwidths

OLS	DV: Number of Days of “Not Good” Mental Health		
State Fixed Effects	Yes	Yes	Yes
Survey Weight	Yes	Yes	Yes
Election	0.321** (0.081)	0.427** (0.088)	0.442** (0.098)
Most likely Democrat	0.184** (0.064)	0.178** (0.064)	0.185** (0.064)
Post‐election × most likely Democrat	0.644** (0.177)	0.574** (0.194)	0.131 (0.216)
Omitted observations (weeks since election)	Within 1 week	Within 2 weeks	Within 3 weeks
*N*	172,789	168,461	164,365
Adjusted *R*‐squared	0.017	0.017	0.016

Abbreviations: BRFSS, Behavioral Risk Factor Surveillance System; DV, dependent variable; OLS, ordinary least squares.

**P* < .1; ***P* < .05.

**Table A9:** Regression Analysis of BRFSS 2024 Poor Mental Health Days by Likely Party and Policy Exposure

OLS	DV: Number of Days of “Not Good” Mental Health
State fixed effects	Yes
Survey weight	Yes
Election	0.207** (0.092)
Most likely Democrat	0.126 (0.078)
Has government insurance	2.273** (0.177) (0.063)
Election × most likely Democrat	0.188 (0.208)
Election × has government insurance	0.209 (0.162)
Most likely Democrat × has government insurance	−0.175 (0.131)
Election × most likely Democrat × has government insurance	1.260** (0.345)
*N*	177,806
Adjusted *R*‐squared	0.017

Abbreviations: BRFSS, Behavioral Risk Factor Surveillance System; DV, dependent variable; OLS, ordinary least squares.

**P* < .1; ***P* < .05.
